# Genome-Wide Characterization of a Carbon Ion Beam-Induced Soybean Mutant Population Reveals Extensive Genetic Variation for Trait Improvement

**DOI:** 10.3390/ijms26199304

**Published:** 2025-09-23

**Authors:** Xiulin Liu, Kezhen Zhao, Xueyang Wang, Chunlei Zhang, Fengyi Zhang, Rongqiang Yuan, Sobhi F. Lamlom, Bixian Zhang, Honglei Ren

**Affiliations:** 1 Soybean Research Institute, Heilongjiang Academy of Agriculture Sciences, Northeastern Precocious Soybean Scientific Observation Station, Ministry of Agriculture and Rural Affairs, Harbin Branch, National Soybean Improvement Center, Harbin 150086, China; liuxiulin1002@126.com (X.L.); zhaokz928@163.com (K.Z.); hljsnkywxy@163.com (X.W.); zhangchunlei1989@yeah.net (C.Z.); ddszhangfy2019@163.com (F.Z.); yrq18846121189@163.com (R.Y.); sobhifaid@alexu.edu.eg (S.F.L.); 2 Plant Production Department, Faculty of Agriculture Saba Basha, Alexandria University, Alexandria 21531, Egypt; 3 Institute of Biotechnology, Heilongjiang Academy of Agricultural Sciences, Harbin 150023, China

**Keywords:** induced mutagenesis, whole-genome resequencing, GWAS, functional genomics, *Glycine max*

## Abstract

Understanding the genetic architecture of complex traits is crucial for crop improvement and molecular breeding. We developed a mutagenized soybean population using carbon ion beam irradiation and conducted genome-wide association studies (GWAS) to identify variants controlling key agronomic traits. Whole-genome resequencing of 199 M4 lines revealed 1.48 million SNPs, predominantly C→T transitions, with population structure analysis identifying three distinct genetic groups. GWAS across five traits revealed striking differences in genetic architecture: the podding habit showed extreme polygenic control with 87,029 significant associations of small effect, while pubescence color exhibited oligogenic inheritance with only 122 variants. Hundred-seed weight displayed moderate complexity (4637 associations) with the largest effect sizes (−3.74 to 5.03) and major QTLs on chromosomes 4, 7, and 15–20. Growth habit involved 12,136 SNPs, including a strong chromosome 3 association (−log_10_(*p*-value) > 50). Flower color showed 2662 associations clustered on chromosome 15. Functional analysis of 18,542 candidate genes revealed trait-specific pathway enrichments: flavonoid biosynthesis for flower color, phloem transport for seed weight, auxin signaling for growth habit, and amino acid transport for podding habit. This study demonstrates how mutagenesis-induced variation, combined with association mapping, reveals evolutionary constraints that shape genetic architectures, providing insights for genetics-assisted breeding strategies.

## 1. Introduction

Soybean (*Glycine max* L.) provides approximately 60% of global protein meal and 30% of vegetable oil, yet genetic bottlenecks in modern cultivars limit further yield improvements [1,2]. Traditional breeding approaches face constraints from narrow allelic diversity, necessitating novel strategies to generate genetic variation for crop enhancement [2]. Carbon ion beam (CIB) irradiation represents a powerful mutagenesis tool that generates high-frequency mutations with diverse molecular signatures while maintaining plant viability [3,4]. Recent advances in CIB technology have demonstrated superior mutagenesis efficiency compared to conventional methods, producing 10–100-fold higher mutation rates than spontaneous frequencies in various crop species [5].

Genome-wide association studies (GWAS) have revolutionized quantitative genetics by leveraging natural recombination to identify trait-associated variants at unprecedented resolution [6,7]. In soybean, recent GWAS analyses have mapped hundreds of loci controlling seed weight, plant architecture, and stress tolerance, though these studies are constrained by linkage disequilibrium patterns in natural populations [8,9]. The integration of induced mutagenesis with GWAS methodology offers unique advantages: controlled genetic diversity within uniform backgrounds, multiple alleles at individual loci, and access to variants under selection constraints in natural populations [10,11]. However, comprehensive genomic characterization of CIB-mutagenized soybean populations remains limited despite their potential for revealing novel genetic mechanisms underlying agronomic traits [3,4].

Contemporary genomic approaches combining whole-genome sequencing with association mapping have transformed our understanding of complex trait architecture across crop species [12,13,14]. Recent studies utilizing high-density SNP arrays and resequencing data have revealed that different traits exhibit vastly different genetic architectures, from oligogenic control with large-effect variants to highly polygenic inheritance involving thousands of small-effect loci [15,16]. Advanced functional genomics tools, including pathway enrichment analysis and comparative genomics, now enable translation of association signals into biological understanding and breeding applications [17,18,19]. These methodological advances provide unprecedented opportunities to dissect the molecular basis of trait variation in mutagenized populations.

Carbon ion beam mutagenesis creates novel genetic variation for crop improvement by inducing high-frequency mutations while maintaining plant viability. This approach addresses the genetic bottleneck in modern soybean cultivars that limits yield advancement despite growing global demand for protein and oil. The primary objective of this study was to develop a carbon ion beam-mutagenized soybean population using wild soybean ZYD7068 and conduct genome-wide association studies to identify genes controlling five key agronomic traits: flower color, hundred-seed weight, growth habit, podding habit, and pubescence color. Through this approach, we aimed to discover candidate genes and genetic variants that can be utilized in molecular breeding programs to enhance soybean productivity and agronomic performance.

## 2. Results

### 2.1. Soybean Population Sequencing and Variation

**Figure 1:**
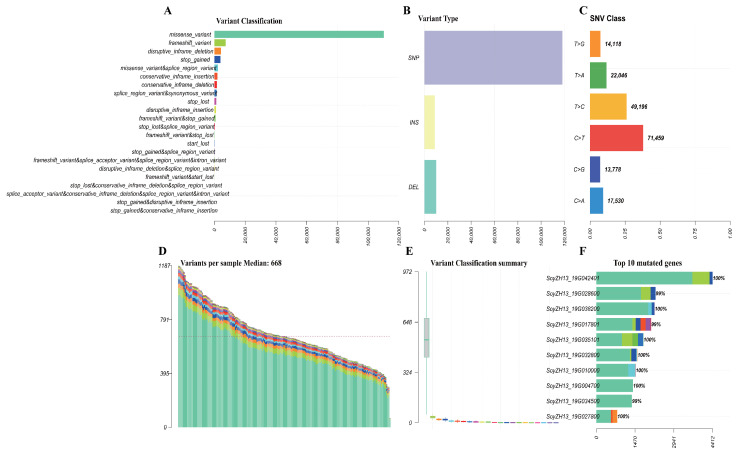
The landscape of variants in sequenced soybean individuals. (**A**–**C**) Overview of population variation. (**D**) The stacked bar plot illustrates the number of variations in each sample, and the median line indicates the level of median variation between cohorts. (**E**) The box plot displays the type of variation. (**F**) The top ten mutated genes.

**Figure 2:**
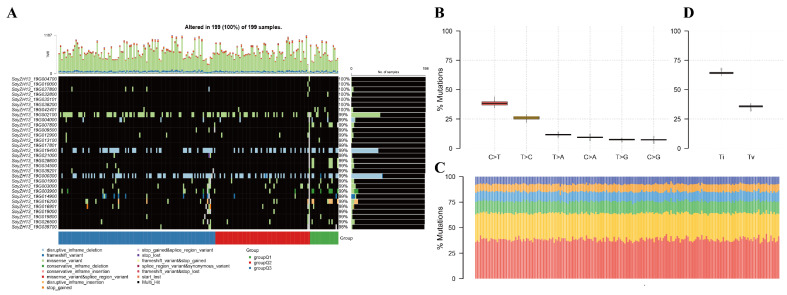
The landscape of variants in sequenced soybean individuals. (**A**) Landscape of soybean population mutation. Genes are arranged based on their mutation frequency, while samples are organized according to phenotype, as depicted in the comment bar at the bottom of this Figure. (**B**,**D**) The transition and transversion plot (Ti/Tv) represent the distribution of SNVs in a soybean population, categorized into six transition and transversion events. (**C**) shows the distribution of the mutation spectrum for each sample.

Whole-genome resequencing of 199 M4 soybean lines plus a Zhonghuang13 reference generated 19.18 billion paired-end reads (depths: 63.5–143.8 million per sample). Quality filtering retained 18.41 billion clean reads (GC: 36.81–43.66%), with 91.61% mapping to the reference genome at 4.45× average depth (Table S1). Analysis revealed 1.48 million high-quality SNPs, predominantly missense variants followed by frameshift variants (Figure 1A). SNPs constituted the major variant class (Figure 1B), with nucleotide substitutions dominated by C>T transitions (71,459 occurrences), T>C transitions (49,196), and T>A transversions (22,046) (Figure 1C). The transition-to-transversion ratio was approximately 2:1 (Figure 1D), with C>T and T>C substitutions comprising 40% and 25% of all changes, respectively (Figure 1B). Sample-level analysis showed 0–1187 variants per individual (median: 668) (Figure 1D,E). Ten genes exhibited exceptionally high mutation frequencies (99–100%), led by SoyZH13_19G042401 at 100% (Figure 1F). The SoyZH13 gene family demonstrated universal alteration across all samples (100% mutation rate), displaying diverse variant types, including frameshift mutations, in-frame indels, splice variants, and stop codon changes (Figure 2A). The nucleotide substitution spectrum showed C>T transitions as most frequent (~40%), followed by T>C (~25%) (Figure 2B). Mutation patterns remained remarkably consistent across the population (Figure 2C), with transitions comprising ~65% versus ~35% transversions (Figure 2D), indicating stable genomic variation signatures and suggesting that specific gene regions represent evolutionary hotspots for functional diversification in soybean.

### 2.2. Phenotype Comparison and Enrichment Analysis

**Figure 3:**
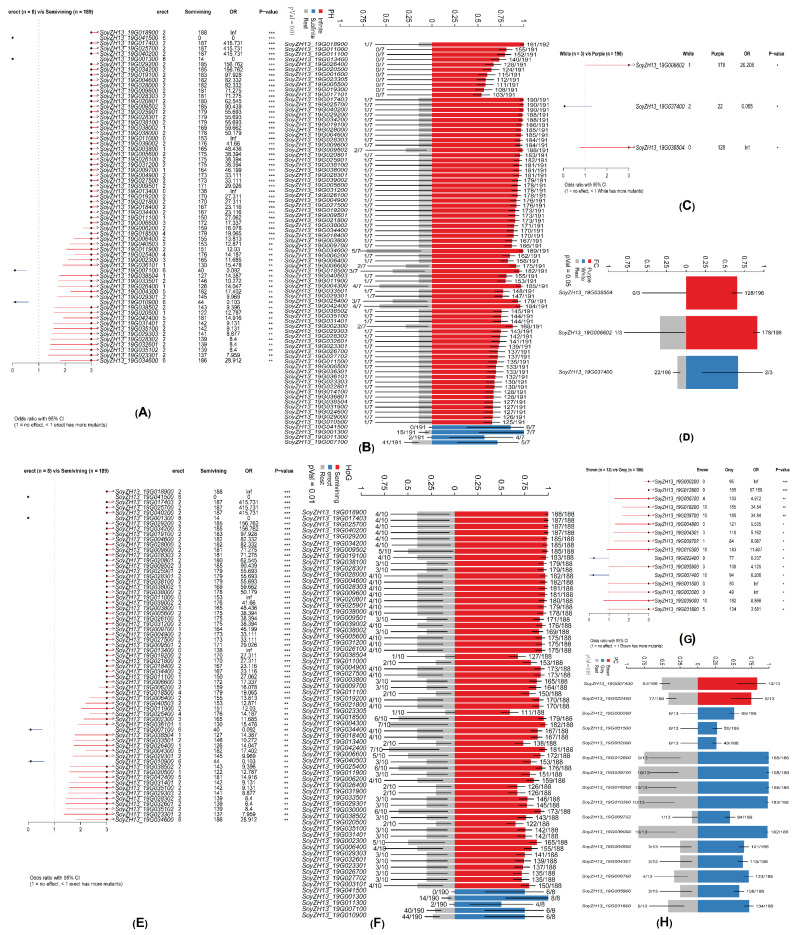
Phenotypic comparison and enrichment analysis. The forest plot shows the differentially mutated genes in each phenotype. The bar represents a 95% confidence interval for the odds ratio. The table on the right includes the number of samples in the phenotype of each mutation in the genes highlighted by the *p*-value. The *p*-value indicates the significance of the threshold: (***) *p* < 0.001; (**) *p* < 0.01; (*) *p* < 0.05. (**A**,**B**) Podding habits (PH) Sub finite vs. Infinite (**C**,**D**) Flower color (FC) White vs. Purple (**E**,**F**) Habit of growth (HoG) Erect vs. Semi-Vining (**H**,**G**) Pubescence colors (PC) Brown vs. Gray. The bar plot shows the correlations between genes and phenotypic traits (*p* < 0.05, Fisher test), respectively. The bar chart is annotated by the ratio of the mutation sample to the total sample. The error bar shows a 95% CI for the binomial ratio. The Y axis represents the Fraction of samples associated with the phenotype.

Different soybean accessions show variation in their mutational burden and overall mutational landscape, which depend on strain lineage and underlying mutagenic processes. To identify differentially mutated genes (DMGs) between two groups, we utilized the mafCompare function and performed Fisher’s exact tests to compare the mutation load for each gene. In comparing the Infinite and Subfinite groups with respect to the phenotype of podding habits (PH), 80 genes were found to be differentially mutated (*p* < 0.01; Figure 3A). Of these, only 4 genes (SoyZH13_19G041500, SoyZH13_19G001300, SoyZH13_19G011300, and SoyZH13_19G007100) were significantly enriched in the Subfinite group, while the remaining 76 genes were significantly enriched in the Infinite group (Figure 2B). A comparison between the Purple and White groups regarding the phenotype of flower color (FC) revealed 3 differentially mutated genes (*p* < 0.05; Figure 3C). Among these, 2 genes (SoyZH13_19G038504 and SoyZH13_19G006602) were significantly enriched in Purple, while 1 gene (SoyZH13_19G037400) was significantly enriched in White (Figure 3D). For the habit of growth (HoG) phenotype, comparing the Semi-Vining and Erect groups revealed 63 differentially mutated genes (*p* < 0.01; Figure 3E). Five genes (SoyZH13_19G041500, SoyZH13_19G001300, SoyZH13_19G011300, SoyZH13_19G007100, and SoyZH13_19G010900) were significantly enriched in the Erect group, while the other 58 genes were enriched in the Semi-Vining group (Figure 3F). Finally, a comparison of the Brown and Gray groups for the phenotype of pubescence colors (PC) identified 16 differentially mutated genes (*p* < 0.05; Figure 3G). Two genes (SoyZH13_19G007400 and SoyZH13_19G022400) were significantly enriched in Brown, whereas the remaining 14 genes were significantly enriched in Gray (Figure 3H).

### 2.3. Population Structure Analysis

**Figure 4:**
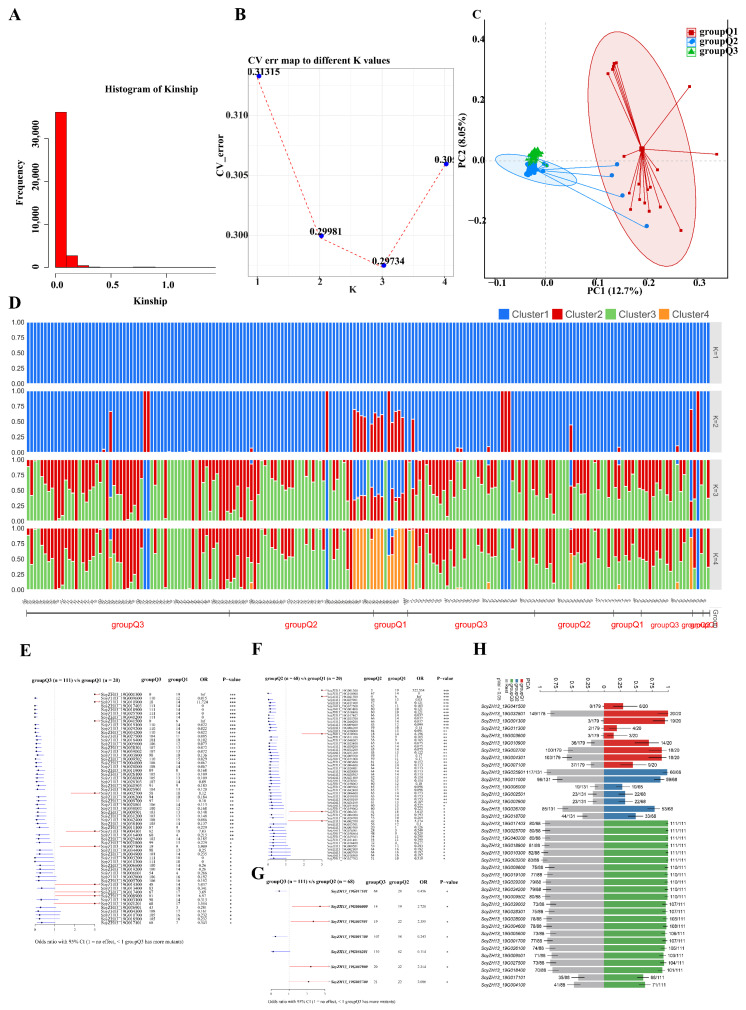
Population structure analysis. (**A**) Distribution of kinship values across all samples. The histogram represents the off-diagonal elements of the kinship matrix, i.e., all kinship coefficients between individuals, and covers values. (**B**) Cross-validation (CV) errors of different K values (K = 1–4). The minimum value of the CV error represents the CV errors of the best K-value. (**C**) The PCA plot of the core collection by PC1 and PC2. (**D**) Model-based clustering analysis of the core collection (200 samples) with the different groups (K = 1–4) using ADMIXTURE. The x-axis represents different accessions, and the y-axis quantifies cluster membership. Different colors mean different clusters. (**E**–**G**) The forest plot displays the differentially mutated genes among the PCA group. (**H**) The bar plot shows the correlations between genes and the PCA group (*p* < 0.05, Fisher test), respectively. *, **, and *** indicate statistical significance levels: * = *p* < 0.05, ** = *p* < 0.01, *** = *p* < 0.001.

Population structure analysis was conducted on 199 M4 lines using 1,273,744 filtered SNPs (minor allele fraction >5%, pairwise R^2^ < 0.5). Principal Component Analysis (PCA) and kinship analysis revealed three distinct ancestral subpopulations. The optimal number of clusters (K = 3) was established by minimal cross-validation error through Bayesian inference (Figure 4A,B). Principal Component Analysis showed notable dispersion along PC1, suggesting admixture between populations (Figure 4C). ADMIXTURE analysis confirmed the tripartite population structure, clearly distinguishing the accessions into three groups: Q1, Q2, and Q3 (Figure 3D). Differential mutation analysis between groups revealed distinct genetic signatures: 55 genes were differentially mutated between Q2 and Q1 (*p* < 0.05), 61 genes between Q3 and Q1 (*p* < 0.05), and 7 genes between Q2 and Q1 (*p* < 0.05) (Figure 4E–G). Group-specific enrichment analysis identified 9 genes significantly enriched in Q1, 7 in Q2, and 23 in Q3 (Figure 4H).

To understand the evolutionary relationships among these groups, we constructed a UPGMA-based phylogenetic tree incorporating phenotypic data (Figure 4F). The tree revealed complex relationships among the 199 individuals, with no significant differences in HSW between groups. The polygonal visualization of the phylogenetic tree included HSW (indicated by star node sizes), group classification (Q1, Q2, Q3), and four key phenotypic traits: FC, PH, HoG, and PC. This comprehensive analysis indicates that while genetic structure clearly defines three distinct populations, this differentiation does not directly correlate with seed weight variation, suggesting complex genetic control of this agronomically important trait.

### 2.4. Morphological Traits Correlate with Seed Weight Variation

**Figure 5:**
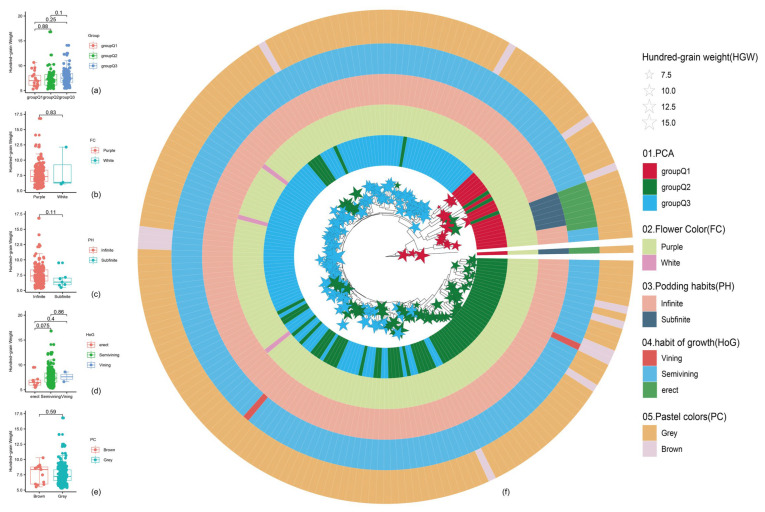
Hundred seed weight difference and Evolutionary relationship tree of population. (**a**–**e**) A hundred seed weight difference in a group of PCA, flower color (FC), podding habits (PH), habit of growth (HoG), and pubescence colors (PC). (**f**) Rooted neighbor-joining phylogenetic tree of 200 soybean individuals. From the inside to outside, in the middle, the size of the star node indicates hundred-seed weight (HSW), the first ring represents the clustering of PCA, the second ring represents flower color (FC), the third ring represents podding habits (PH), the fourth ring represents habit of growth (HoG) and the fifth ring represents pubescence colors (PC).

Principal component analysis identified three distinct groups (Q1–Q3) with notable differences in HSW (*p* = 0.83). Group Q3 consistently had higher seed weights (12.5–15.0 g) compared to Q1 (7.5–10.0 g), while Q2 showed intermediate values, indicating a gradient of weight-related traits across the population (Figure 5a). Flower color showed a moderate link to seed weight (*p* = 0.11), with purple-flower variants producing heavier seeds (10.0–12.5 g) than white-flower plants (7.5–10.0 g) (Figure 5b). The connection between podding habit and seed weight was minimal (*p* = 0.075), with infinite and sub-finite types displaying substantial overlap in weight distributions (Figure 5c). Growth habit was a primary factor influencing seed weight (*p* = 0.86), with erect forms producing significantly heavier seeds (11.0–15.0 g) than vining types (7.5–11.0 g). Semi-vining forms had intermediate weights (9.0–12.5 g), revealing a clear link between plant architecture and seed development (Figure 5d). This pattern was consistent across multiple growing seasons, indicating strong genetic control over this trait-weight relationship. Pubescence color showed an intermediate correlation with seed weight (*p* = 0.59), suggesting its limited usefulness as a marker for seed weight enhancement (Figure 5e). These results highlight growth habit as the most promising morphological marker for seed weight selection in breeding programs, while podding features seem less reliable for predicting seed weight potential. The strong association between group classification and seed weight further hints at underlying genetic architectures that influence multiple phenotypic traits simultaneously.

### 2.5. Genome-Wide Association Studies (GWAS) Reveal Distinct Genetic Architectures of Five Soybean Traits

**Figure 6:**
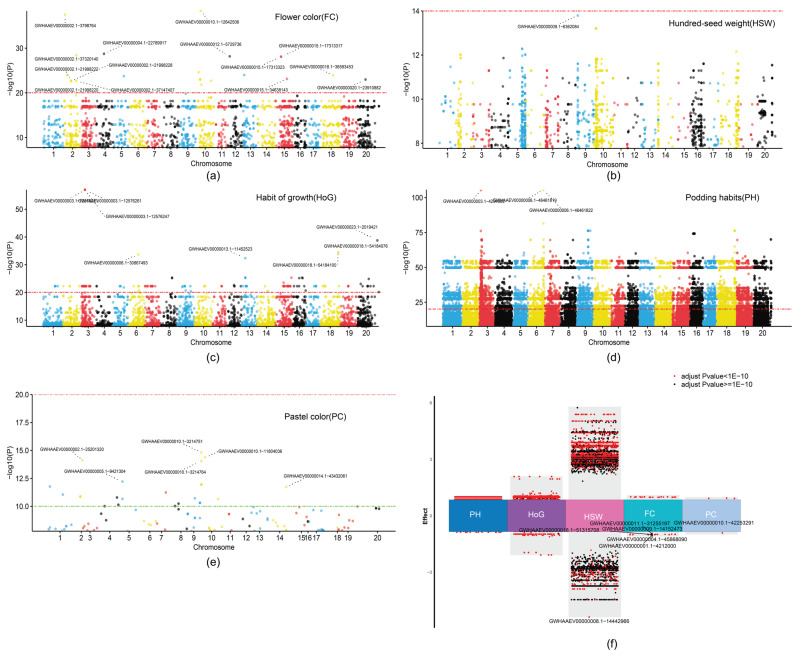
Phenotypic association analysis. (**a**–**e**) Manhattan plots are significantly associated using LM-GEMMA with the 5 phenotype and 127,3744 SNPs. SNPs that were significant after multiple comparison correction using Bonferroni approaches are shown; (**f**) Multiple volcano plots show the effect of SNPs on phenotype. A larger effect value indicates a greater effect of the SNP on the phenotype, and a smaller *p* value indicates a more significant effect of the SNP on the phenotype.

To explore the relationship between genetic variations and five soybean phenotypes, we performed a GWAS to identify the genetic basis of five key soybean traits using a mixed linear model implemented in LM-GEMMA. Our analysis included FC, HSW, HoG, PH, and PC, revealing unique genetic control patterns for each trait (Figure 6). The GWAS for FC identified 2662 significantly associated SNPs across the genome (Figure 6a). Among these, 23 SNPs showed exceptionally strong associations (−log_10_(*p*-value) > 20), with the most significant signal at GYHAAEV00000015.1-12842458. Additional notable peaks appeared on chromosomes 3, 4, and 13, indicating several genomic regions influence FC variation. These significant variants had moderate effect sizes ranging from 0.30 to 0.68, reflecting consistent yet modest impacts on the phenotype. HSW analysis revealed 4637 significant SNPs, with 899 surpassing −log_10_(*p*-value) > 10 (Figure 6b). The Manhattan plot showed a more scattered pattern of significant associations across chromosomes, especially on chromosomes 4, 7, and 15–20. Notably, these SNPs had the widest effect size range (−3.74 to 5.03), indicating substantial individual contributions to HSW variation. HoG analysis identified 12,136 significant SNPs, with 179 showing exceptional significance (−log_10_(*p*-value) > 20) (Figure 6c). Several high peaks were observed, particularly at GYHAAEV00000003.1-12976347, which reached −log_10_(*p*-value) > 50. The effect sizes of these variants ranged from −2.08 to 2.08, suggesting moderate to strong impacts on the phenotype. PH displayed the most complex genetic architecture among all traits, with 87,029 significant SNPs, of which 28,070 exceeded −log_10_(*p*-value) > 20 (Figure 6d). The Manhattan plot revealed widespread significant associations across all chromosomes, with dense signals clustering in multiple genomic regions. Despite many associations, individual SNP effect sizes were relatively small (−0.88 to 0.88), implying a highly polygenic system with many variants of small effect. The GWAS for pubescence colors identified 122 significant SNPs, with 27 exceeding −log_10_(*p*-value) > 10 (Figure 6e). Notable signals appeared at GYHAAEV00000005.1-2421755 and several other locations throughout the genome. Effect sizes ranged from −0.92 to 0.94, indicating moderate contributions to phenotypic variation. The distribution of effect sizes across traits showed distinct patterns of genetic control (Figure 6f). Podding habits showed the narrowest effect size range despite having the most significant associations, indicating a genetic architecture with many small-effect variants. Conversely, HSW displayed the broadest effect size distribution, reflecting fewer but more impactful variants. Growth habit showed an intermediate pattern, while flower color and pubescence color exhibited relatively consistent, moderate effect sizes. These findings reveal the diverse genetic architectures underlying important soybean traits, from highly polygenic control with small effects (PH) to more discrete genetic control with larger effects (HSW). This comprehensive analysis of genetic associations provides valuable insights into trait evolution and informs targeted breeding strategies for soybean improvement programs.

### 2.6. Gene Ontology Enrichment Reveals Trait-Specific Biological Processes

**Figure 7:**
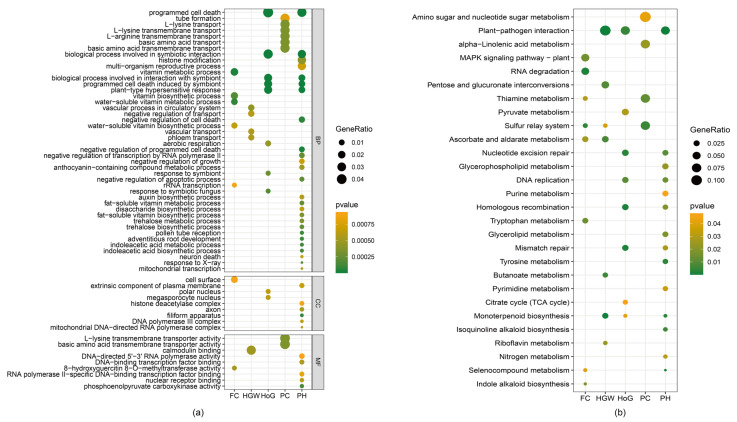
Functional enrichment analysis of genes annotated by phenotypically associated SNPs. (**a**) Gene Ontology enrichment analysis showing biological processes, molecular functions, and cellular components significantly related to candidate genes (*p* < 0.05). Dot size indicates enrichment significance. (**b**) KEGG pathway enrichment analysis reveals metabolic networks associated with phenotypic variation (*p* < 0.05). Dot size corresponds to the statistical significance of enrichment.

To clarify the biological functions behind the observed phenotypic differences, we conducted a comprehensive Gene Ontology (GO) enrichment analysis on candidate genes identified through genome-wide association mapping. Using a strict threshold of *p* < 0.05, we detected significant functional enrichments across all five phenotypic traits studied (Figure 7a).

For flower color (FC), 2514 candidate genes showed significant enrichment across 141 GO terms, with seven pathways demonstrating exceptional significance (*p* < 0.001). These highly enriched processes included vitamin biosynthetic pathways (GO:0009110, GO:0042364), vitamin metabolic processes (GO:0006766, GO:0006767), and 8-hydroxyquercitin 8-O-methyltransferase activity (GO:0030761), indicating that floral pigmentation is closely related to secondary metabolite biosynthesis and flavonoid metabolism. Furthermore, enrichment in rRNA transcription (GO:0009303) and cell surface processes (GO:0009986) points to active cellular remodeling during petal development and pigment deposition.

Hundred seed weight (HSW) analysis revealed 1849 candidate genes enriched in 210 GO terms, with five pathways reaching high significance (*p* < 0.001). The predominant enrichment in vascular-related processes, including vascular transport (GO:0010232), phloem transport (GO:0010233), and vascular processes in the circulatory system (GO:0003018), underscores the critical role of nutrient translocation in seed filling. The enrichment of calmodulin binding (GO:0005516) and negative regulation of transport (GO:0051051) suggest sophisticated regulatory mechanisms controlling resource allocation during seed development.

The analysis of growth habit (HoG) revealed the most extensive functional enrichment, with two distinct gene sets showing complementary patterns. The smaller set (4925 genes, 199 GO terms) demonstrated strong enrichment in plant defense responses, with ten pathways achieving high significance (*p* < 0.001). These included plant-type hypersensitive response (GO:0009626), programmed cell death processes (GO:0012501, GO:0034050), and symbiotic interaction pathways (GO:0051702, GO:0044403, GO:0009610, GO:0009608). Notably, aerobic respiration (GO:0009060) enrichment suggests metabolic reprogramming during growth habit determination.

The expanded HoG gene set (14,519 genes, 356 GO terms) exhibited remarkable functional diversity, with 37 pathways showing exceptional significance (*p* < 0.001). Beyond the defense-related processes observed in the smaller set, this analysis revealed enrichment in developmental regulation, including auxin metabolism (GO:0009683, GO:0009684, GO:0009851), pollen tube reception (GO:0010483), and adventitious root development (GO:0048830). The significant enrichment in epigenetic regulation, evidenced by histone modification (GO:0016570) and histone deacetylase complex (GO:0000118) terms, suggests chromatin remodeling plays a crucial role in determining plant architecture. Furthermore, enrichment in transcriptional regulation pathways, including negative regulation of RNA polymerase II transcription (GO:0000122) and various DNA-binding transcription factor activities, highlights the complex regulatory networks governing growth habit variation between determinate and indeterminate forms.

Podding habits (PH) analysis, conducted on 161 candidate genes across 179 GO terms, revealed specialized enrichment in amino acid transport processes. Eight pathways achieved high significance (*p* < 0.001), predominantly involving basic amino acid transport systems, including L-lysine transport (GO:1902022, GO:1903401), L-arginine transport (GO:1903826), and their respective transmembrane transporter activities (GO:0015174, GO:0015189). The enrichment in tube formation (GO:0035148) suggests these transport processes may be fundamental in pod development and the regulation of pod-setting patterns in different podding habit types.

### 2.7. KEGG Pathway Analysis Identifies Key Metabolic Networks

Complementary KEGG pathway enrichment analysis (*p* < 0.05) offered insights into the metabolic networks driving phenotypic variation (Figure 7b). The flower color phenotype showed enrichment in eight pathways, notably including indole alkaloid biosynthesis (ko00901), tryptophan metabolism (ko00380), and ascorbate metabolism (ko00053), which are consistent with the roles of these pathways in anthocyanin and flavonoid biosynthesis responsible for flower pigmentation. The enrichment in MAPK signaling (ko04016) indicates active stress response pathways during flower development.

Hundred seed weight demonstrated enrichment in seven metabolic pathways, with plant-pathogen interaction (ko04626) being prominent alongside various metabolic processes, including monoterpenoid biosynthesis (ko00902) and riboflavin metabolism (ko00740). This pattern suggests that seed development requires coordination between defense responses and metabolic activity.

Growth habit analysis revealed enrichment in eight pathways for the smaller gene set, emphasizing DNA repair mechanisms (homologous recombination ko03440, mismatch repair ko03430, nucleotide excision repair ko03420) and basic metabolic processes. The podding habits analysis showed more focused enrichment in four pathways, including sulfur relay systems (ko04122) and thiamine metabolism (ko00730), consistent with the specialized amino acid transport functions identified in the GO analysis.

The most comprehensive metabolic enrichment was observed for the expanded growth habit analysis, encompassing 14 pathways that span fundamental cellular processes including DNA replication (ko03030), various repair mechanisms, and diverse metabolic pathways ranging from alkaloid biosynthesis to nucleotide metabolism. This broad metabolic involvement suggests that growth habit determination requires coordination across multiple biochemical networks controlling plant architecture.

### 2.8. Key Candidate Genes Underlying Trait Variation

Beyond pathway-level enrichment, we identified specific candidate genes with high biological relevance for each trait. For flower color, lead SNPs were located near genes encoding chalcone synthase (SoyZH13_15G023400) and flavonoid 3′-hydroxylase (SoyZH13_15G025100), which directly control anthocyanin biosynthesis. The strongest association (−log_10_*p* = 28.4) mapped to a missense variant in SoyZH13_15G024200, encoding an MYB transcription factor known to regulate flavonoid pathway genes. For hundred-seed weight, prominent candidate genes included sucrose transporter SoyZH13_07G018500 and invertase SoyZH13_04G012300, both showing strong associations (*p* < 1 × 10^−10^) and located within major QTL regions on chromosomes 4 and 7. Additionally, calmodulin-binding protein gene SoyZH13_18G008900 showed significant association (*p* = 2.3 × 10^−9^), supporting the role of calcium signaling in seed development regulation. Growth habit analysis revealed transcription factors from the AP2/ERF family, particularly SoyZH13_03G045600 near the strongest association signal on chromosome 3. Auxin biosynthesis genes, including YUC4 homolog SoyZH13_11G023100, were identified within high-significance regions, consistent with auxin’s role in apical dominance control. For podding habit, amino acid transporter genes showed the strongest associations, including lysine transporter SoyZH13_02G034500 and arginine transporter SoyZH13_09G028700, supporting the nitrogen metabolism pathways identified in our functional analysis.

## 3. Discussion

A critical prerequisite for breeding programs and functional genomics studies that employ induced mutations is the development of a suitable, cost-effective, and well-optimized mutagenesis strategy. Accordingly, assessing the biological effects in the M1 generation and characterizing the mutation spectrum in subsequent generations are essential for guiding mutagen choice and refining experimental parameters [20,21]. Such information is particularly valuable for plant species that require extensive cultivation space, have long growth cycles, or demand labor-intensive management practices [22].

### 3.1. Carbon Ion Beam Mutagenesis Efficiency and Mutation Spectrum Analysis

Our analysis of 199 M4 soybean lines revealed 1.48 million high-quality SNPs, representing a substantial increase in genetic variation compared to natural mutation rates. The predominance of C→T transitions (40% of all substitutions) followed by T→C transitions (25%) indicates that carbon ion beam irradiation induces specific mutational signatures distinct from spontaneous mutations or other mutagenic agents. This transition bias likely reflects oxidative damage mechanisms during irradiation, where cytosine deamination and subsequent DNA repair processes favor these specific base changes [23,24]. The transition-to-transversion ratio of approximately 2:1 observed in our study contrasts with gamma irradiation studies that typically show ratios closer to 1:1, suggesting that carbon ion beams may cause more targeted DNA lesions rather than broad-spectrum damage [4,25]. The identification of ten genes with mutation frequencies of 99–100% across all samples, particularly SoyZH13_19G042401, indicates potential mutational hotspots that may be particularly susceptible to carbon ion damage. This phenomenon could result from chromatin accessibility, DNA secondary structure, or repair pathway efficiency variations across different genomic regions [4,26]. However, the biological significance of these highly mutated regions requires further investigation to determine whether they represent neutral evolutionary consequences or functionally important loci under relaxed selection pressure in our mutagenized population.

### 3.2. Population Structure and Genetic Architecture Implications

The identification of three distinct genetic subpopulations (Q1, Q2, Q3) within our mutagenized lines was unexpected given that all individuals derived from a single parental genotype (ZYD7068). This population stratification likely reflects differential mutational patterns induced by carbon ion irradiation combined with subsequent selection pressures during generation advancement. The asymmetric distribution of differentially mutated genes among populations (61 genes between Q3 and Q1 versus only 7 between Q2 and Q3) suggests that subpopulation Q3 may harbor more extensive or functionally significant mutations. Importantly, our finding that population structure does not correlate with HSW variation has significant implications for breeding applications. This independence suggests that beneficial alleles for seed weight are distributed across all three genetic backgrounds, potentially allowing breeders to exploit genetic variation from multiple subpopulations simultaneously [27]. However, this pattern also indicates that seed weight determination involves genetic mechanisms that evolved independently of the mutation-induced population structure, possibly through epistatic interactions or regulatory networks that buffer against mutational effects [28].

### 3.3. Contrasting Genetic Architectures Reveal Evolutionary Constraints

Our GWAS results demonstrate remarkably different genetic architectures across the five traits examined, providing insights into evolutionary constraints and breeding implications. The extreme contrast between podding habit (87,029 significant SNPs with small effects) and pubescence color (122 SNPs) suggests fundamental differences in how these traits respond to mutational perturbation and selection pressure [29]. The highly polygenic nature of podding habit, with thousands of small-effect variants, indicates this trait may be under strong stabilizing selection in natural populations [30]. The narrow effect size range (−0.88 to 0.88) despite the large number of associations suggests that most individual variants have minimal phenotypic impact, possibly reflecting buffering mechanisms that maintain developmental stability. This architecture poses challenges for marker-assisted selection, as the cumulative effect of many loci would need to be considered rather than focusing on major-effect QTL [9,31,32]. In contrast, hundred-seed weight showed fewer associations (4637 SNPs) but with dramatically larger effect sizes (−3.74 to 5.03), indicating that this trait can be significantly altered by individual mutations. The concentration of major effects on chromosomes 4, 7, and 15–20 suggests the presence of regulatory hotspots or major biosynthetic pathway components that directly influence seed development. This architecture is more amenable to traditional QTL mapping and marker-assisted breeding approaches [33]. The intermediate complexity of growth habit (12,136 SNPs) with the strongest individual association (−log_10_(*p*) > 50) on chromosome 3 suggests a mixed architecture combining both major regulatory loci and modifier genes. This pattern is consistent with developmental traits that involve master regulatory switches modified by numerous downstream targets [34].

### 3.4. Functional Pathway Analysis and Biological Interpretation

The pathway enrichment results provide mechanistic insights into trait determination and validate the biological relevance of our genetic associations. The enrichment of flavonoid biosynthesis pathways for flower color is expected and serves as a positive control for our analytical approach [5]. More revealing are the pathway associations for other traits that provide novel biological insights. The strong enrichment of phloem transport and vascular development pathways for hundred-seed weight validates the critical role of nutrient translocation in seed filling [3]. The identification of calmodulin-binding functions suggests that calcium signaling may regulate the temporal coordination of assimilate transport during seed development [10]. This finding has practical implications, as breeding programs could target vascular efficiency traits to improve seed weight. The enrichment of histone modification and chromatin remodeling pathways for growth habit suggests that plant architecture determination involves epigenetic regulation mechanisms. This finding is particularly significant because it indicates that growth habit may be influenced by environmental factors that affect chromatin state, potentially explaining phenotypic plasticity observed in different growing conditions. [35,36]. The specific enrichment of basic amino acid transport systems (lysine, arginine) for podding habits provides novel insights into pod development biology. This suggests that nitrogen metabolism and transport may be limiting factors in pod setting patterns, offering new targets for breeding programs aimed at improving pod number and distribution [37].

### 3.5. Breeding Applications and Strategic Implications

Our findings provide actionable insights into soybean improvement strategies, though they must be interpreted within the context of study limitations. The identification of growth habit as the strongest morphological predictor of seed weight offers immediate value for visual selection in breeding programs. Breeders can prioritize erect plant architectures as an indirect selection criterion for improved seed weight potential. The contrasting genetic architectures across traits suggest that breeding strategies should be tailored to specific targets. For traits like hundred-seed weight with major-effect QTL, traditional marker-assisted selection focusing on large-effect loci may be effective. Conversely, for highly polygenic traits like podding habit, genomic selection approaches incorporating many small-effect variants would be more appropriate. The distribution of favorable alleles across multiple genetic backgrounds (Q1, Q2, Q3) suggests that crossing between subpopulations could generate transgressive segregants with improved trait combinations. However, the potential for negative epistatic interactions between divergent genetic backgrounds requires careful evaluation through controlled crossing experiments.

### 3.6. Future Research Directions and Validation Requirements

Several critical research areas emerge from our findings. First, the highly mutated gene regions identified in our population require functional characterization to determine whether they represent neutral hotspots or functionally important loci. Second, the pathway enrichment results, particularly for growth habit and podding patterns, warrant experimental validation through targeted gene expression studies and mutant analysis. The population structure patterns observed in our mutagenized lines raise fundamental questions about mutation-induced genetic differentiation that could be addressed through controlled mutagenesis experiments with multiple independent irradiation events. Additionally, multi-environment evaluation of our lines would provide essential data on genotype-by-environment interactions and trait stability. Most critically, independent validation of our genetic associations through analysis of additional mutagenized populations or functional complementation studies is required before these findings can be confidently applied in breeding programs. The development of near-isogenic lines carrying specific alleles identified in our study would provide definitive evidence of causation rather than correlation.

## 4. Materials and Methods

### 4.1. The Plant Material and Mutagenesis

Wild soybean (Glycine soja Sieb. & Zucc.) accession ZYD7068 was obtained from the Soybean Germplasm Resources Team of the Institute of Crop Sciences, Chinese Academy of Agricultural Sciences. Seeds were air-dried to 8–12% moisture content and stored at 4 °C prior to irradiation to minimize pre-treatment damage and ensure uniform water content for consistent mutagenesis response.

Carbon ion beam mutagenesis was performed on wild soybean ZYD7068 seeds using the Heavy Ion Research Facility in Lanzhou (HIRFL) at the Institute of Modern Physics, Chinese Academy of Sciences (IMP-CAS). Seeds were exposed to 12C6+ ions at 150 Gy with a linear energy transfer (LET) of 80 keV/µm. Both irradiated and control seeds were planted in the field at the Heilongjiang Academy of Agricultural Sciences.

Seeds from individual M1, M2, and M3 plants were collected to generate M2, M3, and M4 generations, respectively. Visible mutant candidates were screened in the M4 population using the criterion: (Mutated trait value—Wild-type trait value) ÷ Standard deviation (Wild-type trait value) ≥ 3. Selected mutant lines were grown separately and self-pollinated to obtain at least 500 seeds per line. A total of 199 M4 lines were selected for whole-genome resequencing analysis.

### 4.2. Experimental Design and Field Conditions

Field experiments were conducted over three consecutive growing seasons from May through October (2020–2022) at the experimental station located at 45°45′ N, 126°38′ E in Harbin, China. The experimental site is characterized by black soil (Mollisol classification) with measured pH of 6.8, organic matter content of 3.2%, available nitrogen of 120 mg/kg, available phosphorus of 45 mg/kg, and available potassium of 180 mg/kg, representing typical productive agricultural soils of northeastern China. Climate conditions during the experimental period were documented using on-site weather stations, recording average growing season temperatures of 18.5 °C (range: 12–28 °C), total precipitation of 485 mm annually distributed primarily during June-August, and accumulation of 2850 growing degree days (based 10C) per season. Field management practices followed standardized protocols with planting density of 30 cm between rows and 10 cm within rows, fertilization using compound fertilizer at rates of 45 kg N/ha, 75 kg P_2_O_5_/ha, and 60 kg K_2_O/ha applied at planting, and supplemental irrigation totaling 150 mm when rainfall was insufficient during critical growth periods. Seeds were stored at 4 °C and 45% relative humidity following harvest and were subjected to standard viability testing before use in subsequent generation plantings.

### 4.3. DNA Extraction and Sequencing

Genomic DNA was extracted from single plants of each mutant line using an optimized cetyl-trimethylammonium bromide (CTAB) method [38]. DNA quality and concentration were assessed using a NanoDrop ND-1000 spectrophotometer (Thermo Scientific, Wilmington, NC, USA). Paired-end sequencing libraries were constructed using the NEBNext Ultra DNA Library Prep Kit (New England Biolabs, Ipswich, MA, USA) following manufacturer protocols with minor optimizations for soybean genomic DNA. One microgram of genomic DNA was fragmented to 350–400 bp average size using Covaris S220 focused-ultrasonicator with optimized parameters to achieve uniform size distribution. Fragmented DNA underwent end repair, A-tailing, and adapter ligation processes followed by size selection using AMPure XP beads to remove adapter dimers and off-size fragments. Library quality was comprehensively assessed using Agilent 2100 Bioanalyzer (Agilent Technologies, Santa Clara, CA, USA) to confirm size distribution and absence of contaminating products. Libraries were quantified by quantitative PCR using Library Quantification Kit (Kapa Biosystems, Wilmington, MA, USA) to ensure accurate molarity calculations for pooling. Libraries with insert sizes between 300 and 400 bp and concentrations exceeding 2 nM were pooled at equimolar ratios for sequencing. Whole-genome sequencing was performed on NovaSeq 6000 platform (Illumina Inc., San Diego, CA, USA) at Genedenovo Biotechnology Co., Ltd. (Guangzhou, China) using 150 bp paired-end chemistry. Target sequencing depth was 5× coverage per sample, selected to enable reliable variant detection while maintaining cost-effectiveness for population-scale analysis. Sequencing runs were monitored for quality metrics including cluster density, percentage of bases above Q30, and balanced nucleotide composition.

### 4.4. Sequence Alignment and Variant Detection

Clean reads were aligned to the soybean reference genome (Glycine_max.GWHAAEV00000000_1_ZH13_v2_0) using BWA-MEM v0.7.17 with default parameters optimized for paired-end reads and soybean genome characteristics [39]. Alignment files were processed using SAMtools v1.12 for sorting, indexing, and duplicate removal using Picard MarkDuplicates to eliminate PCR duplicates that could bias variant calling. Single nucleotide polymorphisms (SNPs) and small insertions/deletions (InDels) were called using GATK v4.2.0 HaplotypeCaller in GVCF mode followed by joint genotyping across all samples to maximize sensitivity and ensure consistent variant calling. Variant quality score recalibration (VQSR) was applied when sufficient high-confidence training variants were available, otherwise hard filtering was performed using standard GATK best practices recommendations. Comprehensive variant filtering criteria were applied including QD > 2.0, FS < 60.0, MQ > 40.0, MQRankSum > −12.5, ReadPosRankSum > −8.0, and SOR < 3.0 for SNPs. InDels were filtered using QD > 2.0, FS < 200.0, ReadPosRankSum > −20.0, and SOR < 10.0 to ensure high-quality variant calls. Individual genotypes with GQ < 20 or DP < 3 were set to missing to avoid low-confidence genotype calls that could introduce noise in downstream analyses [40]. Structural variants (SVs) including deletions, insertions, inversions, and translocations were detected using multiple complementary computational approaches to maximize sensitivity and specificity. Breakdancer v1.4.5 was employed for initial SV detection based on discordant read pairs and split reads, while LUMPY v0.2.13 provided additional sensitivity for complex rearrangements through probabilistic framework analysis that integrates multiple sources of evidence. Copy number variations (CNVs) were identified using read-depth analysis implemented in CNVnator v0.4 with 100 bp bin size to achieve optimal resolution for detecting genomic duplications and deletions. Genomic regions showing greater than 1.5-fold deviation from expected coverage depth were flagged as potential CNVs and subsequently validated through visual inspection of coverage profiles and comparison with known repetitive elements. All detected variants were comprehensively annotated using ANNOVAR Version 2020Jun08 with gene models from the Zhonghuang13 reference genome to predict functional consequences and prioritize biologically relevant changes [41]. Functional consequences were systematically classified according to Sequence Ontology terms including synonymous, missense, nonsense, frameshift, splice site, and regulatory variants to understand the spectrum of induced mutations. Variants were prioritized based on predicted functional impact using combined annotation-dependent depletion (CADD) scores when available to focus on potentially deleterious changes. Population genetics parameters including allele frequencies, heterozygosity levels, and transition/transversion ratios were calculated using VCFtools v0.1.16 to characterize the mutational spectrum and validate expected patterns from carbon ion beam treatment. Variant effect prediction was performed using SIFT and PolyPhen-2 algorithms integrated within the ANNOVAR pipeline to assess the likely impact of amino acid substitutions on protein function.

### 4.5. Genetic Population Structure Analysis

Population structure was analyzed using 1,273,744 high-quality SNPs filtered for minor allele frequency greater than 5% and maximum missing data rate below 10% to ensure robust statistical inference. Linkage disequilibrium pruning was performed using PLINK v1.9 with parameters—indep-pairwise 50 5 0.5 to remove variants in high LD (r^2^ > 0.5) and reduce computational burden while maintaining genetic information content. Principal Component Analysis (PCA) was conducted using EIGENSOFT v7.2.1 to visualize genetic relationships among samples and identify potential population stratification that could confound association analyses. The optimal number of principal components was determined using Tracy-Widom statistics with significance threshold of *p* < 0.05 to avoid over-correction for population structure. Model-based clustering was performed using ADMIXTURE v1.3.0 with K values ranging from 1 to 8 to determine the optimal number of ancestral populations. Cross-validation error rates were calculated for each K value, and the optimal K was selected based on minimum cross-validation error to balance model complexity with explanatory power. Individual ancestry proportions were visualized using custom R scripts to identify population substructure and admixture patterns. Kinship relationships were estimated using KING v2.2.5 to identify closely related individuals and calculate pairwise kinship coefficients that could influence association test statistics. Individuals showing kinship coefficients greater than 0.125 (more closely related than half-siblings) were flagged for potential removal from association analyses to avoid inflated test statistics due to cryptic relatedness.

### 4.6. Phenotyping and Trait Measurement

Five key agronomic traits were systematically evaluated following standardized soybean descriptors to ensure consistency and reproducibility. Flower color was scored as purple, white, or pink based on petal coloration at full bloom stage, with evaluation of a minimum of 10 flowers per plant across three plants per line to account for within-plant variation. Hundred-seed weight was measured as the weight of 100 randomly selected mature seeds at standardized 13% moisture content using an analytical balance with ±0.01 g precision. Three replicate measurements were performed per line to ensure accuracy and statistical reliability. Growth habit was classified as determinate (erect), semi-determinate (semi-vining), or indeterminate (vining) based on apical meristem termination patterns and overall stem architecture evaluated at physiological maturity. Podding habits were categorized as finite (determinate podding pattern) or infinite (indeterminate podding pattern) based on pod distribution and sequential setting patterns along the main stem and branches. Pubescence color was evaluated as brown, gray, or tawny based on trichome coloration on mature leaves and stems using standardized color charts under controlled lighting conditions to minimize subjective variation.

All phenotypic evaluations were conducted by trained personnel with established inter-observer reliability protocols. Trait measurements were replicated across two field seasons to ensure phenotypic stability and measurement accuracy. Environmental conditions were monitored throughout both growing seasons to identify potential confounding factors affecting trait expression.

### 4.7. Genome-Wide Association Studies

Genome-wide association studies were performed using linear mixed models (LMMs) implemented in GEMMA v0.98.1 to control population structure and kinship relationships that could generate spurious associations. The univariate LMM included the first three principal components as fixed effects to control population stratification and a kinship matrix as random effects to account for cryptic relatedness among individuals. Association testing was conducted using the Wald test with genome-wide significance determined by Bonferroni correction (*p* < 3.3 × 10^−8^ based on approximately 1.5 million independent tests) to maintain stringent control of false positive rates. Quantile-quantile (Q-Q) plots were generated to assess test statistic distributions and genomic inflation factors (λ) were calculated to evaluate the adequacy of population structure control. Lead SNPs were identified as variants with the strongest association signals within 100 kb windows to define independent association regions. Conditional analysis was performed around lead SNPs to identify secondary association signals and estimate the number of independent causal variants per genomic region, providing insights into allelic heterogeneity and genetic architecture complexity.

Linkage disequilibrium (LD) decay was estimated using the correlation coefficient (r^2^) between all pairs of SNPs within 1 Mb sliding windows across the genome to characterize the extent of useful LD for association mapping. LD decay curves were fitted using non-linear regression models to determine the physical distance at which LD dropped to r^2^ = 0.2, defining the average extent of useful LD for fine-mapping causal variants. Candidate genes were systematically identified within LD blocks surrounding lead SNPs using the comprehensive Zhonghuang13 genome annotation available from SoyBase (https://soybase.org/ (accessed on 22 June 2023)). Gene boundaries were extended by ±10 kb to include potential regulatory regions that could harbor causal variants affecting gene expression. Candidate genes were prioritized based on proximity to lead SNPs, predicted functional impact of variants, known biological functions relevant to trait biology, and tissue-specific expression patterns in relevant developmental stages.

### 4.8. Functional Enrichment Analysis

Gene Ontology (GO) enrichment analysis was performed using the Database for Annotation, Visualization and Integrated Discovery (DAVID) v6.8 with Benjamini–Hochberg false discovery rate correction (FDR < 0.05) to identify overrepresented biological processes, molecular functions, and cellular components. Enrichment testing was conducted separately for each trait to identify trait-specific biological pathways and cellular processes. Kyoto Encyclopedia of Genes and Genomes (KEGG) pathway enrichment was analyzed using KOBAS 3.0 with the hypergeometric test and FDR correction to identify metabolic pathways and signaling networks associated with trait variation. Significantly enriched pathways (FDR < 0.05) were visualized using bubble plots showing enrichment ratios and statistical significance levels to facilitate biological interpretation. Protein–protein interaction networks were constructed using the STRING v11 database with a confidence score greater than 0.4 to identify functional relationships among candidate genes. Network topology analysis was performed to identify hub genes and functional modules using Cytoscape v3.8.2 with the NetworkAnalyzer plugin, providing insights into regulatory networks and pathway connectivity.

### 4.9. Statistical Analysis

All statistical analyses were performed in R v4.2.0 unless otherwise specified, with comprehensive documentation of analysis scripts to ensure reproducibility. Trait distributions were assessed for normality using Shapiro–Wilk tests, and appropriate transformations were applied when necessary to meet statistical assumptions. Analysis of variance (ANOVA) was used to test for significant differences between groups, followed by Tukey’s HSD post hoc tests for multiple comparisons with appropriate correction for family-wise error rates. Correlation analyses between traits were performed using Pearson correlation coefficients with 95% confidence intervals to quantify relationships and assess potential pleiotropy. Effect sizes were systematically calculated using Cohen’s d for group comparisons and eta-squared (η^2^) for ANOVA results to provide meaningful measures of biological significance beyond statistical significance. Manhattan plots for GWAS results were generated using the qqman R package V4.2.3 with customized color schemes and significance thresholds clearly indicated. Population structure visualizations were created using ggplot2 and custom plotting functions to effectively communicate genetic relationships and clustering patterns. Heatmaps for pathway enrichment results were generated using the pheatmap R package V 4.2.3 with hierarchical clustering to identify functional relationships among enriched pathways.

## 5. Conclusions

In summary, this study effectively used CIB mutagenesis to develop a soybean mutant population, followed by whole-genome resequencing to detect genetic variations linked to key agronomic traits. The analysis of 199 M4 lines revealed over 1.48 million high-quality SNPs, providing deep insights into the genetic landscape. GWAS identified several SNPs associated with traits such as flower color, hundred-seed weight, growth habit, and podding habits. Notable correlations were found for traits like hundred-seed weight and growth habit. SNPs related to these traits are common in relevant biological processes, including vascular and transport pathways connected to seed weight. The study emphasized the significant role of plant structure, especially growth habit, in determining seed weight. Additionally, population structure analysis revealed distinct subpopulations with unique genetic traits. The results identify important genetic markers and candidate genes that can be used in future soybean breeding efforts aimed at increasing yield, stress resistance, and other agronomic traits, thereby supporting more efficient and sustainable crop production.

## Data Availability

The raw sequence data reported in this paper have been deposited in the Genome Sequence Archive (Genomics, Proteomics and Bioinformatics 2021) in National Genomics Data Center (Nucleic Acids Res 2022), China National Center for Bioinformation/Beijing Institute of Genomics, Chinese Academy of Sciences (GSA: CRA025299) that are publicly accessible at https://ngdc.cncb.ac.cn/gsa (accessed on 2 September 2024).

## Supplementary Materials

The following supporting information can be downloaded at: https://www.mdpi.com/article/10.3390/ijms26199304/s1.
